# Virtual Experience Toolkit: An End-to-End Automated 3D Scene Virtualization Framework Implementing Computer Vision Techniques

**DOI:** 10.3390/s24123837

**Published:** 2024-06-13

**Authors:** Pau Mora, Clara Garcia, Eugenio Ivorra, Mario Ortega, Mariano L. Alcañiz

**Affiliations:** Research in Human-Centred Technology University Research Institute, Universitat Politècnica de València, 46022 Valencia, Spain

**Keywords:** 3D scene understanding, indoor scenes, virtual reality (VR), ScanNet, scene reconstruction

## Abstract

Virtualization plays a critical role in enriching the user experience in Virtual Reality (VR) by offering heightened realism, increased immersion, safer navigation, and newly achievable levels of interaction and personalization, specifically in indoor environments. Traditionally, the creation of virtual content has fallen under one of two broad categories: manual methods crafted by graphic designers, which are labor-intensive and sometimes lack precision; traditional Computer Vision (CV) and Deep Learning (DL) frameworks that frequently result in semi-automatic and complex solutions, lacking a unified framework for both 3D reconstruction and scene understanding, often missing a fully interactive representation of the objects and neglecting their appearance. To address these diverse challenges and limitations, we introduce the Virtual Experience Toolkit (VET), an automated and user-friendly framework that utilizes DL and advanced CV techniques to efficiently and accurately virtualize real-world indoor scenarios. The key features of VET are the use of ScanNotate, a retrieval and alignment tool that enhances the precision and efficiency of its precursor, supported by upgrades such as a preprocessing step to make it fully automatic and a preselection of a reduced list of CAD to speed up the process, and the implementation in a user-friendly and fully automatic Unity3D application that guides the users through the whole pipeline and concludes in a fully interactive and customizable 3D scene. The efficacy of VET is demonstrated using a diversified dataset of virtualized 3D indoor scenarios, supplementing the ScanNet dataset.

## 1. Introduction

Virtual reality (VR) has transformed into a groundbreaking technology, impacting a wide array of fields ranging from entertainment and gaming to healthcare, education, and beyond [1,2]. While VR technology itself is fascinating, it is the content that users interact with—the virtualized environments and objects—that truly defines the user’s experience. Therefore, in the context of VR, the concept of virtualization is fundamental, acting as the critical foundation for crafting these interactive and immersive worlds.

Virtualization enables the digital replication of real-world scenes, objects, and interactions within a virtual space, providing the heightened realism that is crucial for an immersive experience [3,4]. The more accurate and detailed the virtualization, the more convincing the virtual environment becomes. This not only enhances the user’s sense of immersion but also has practical implications, such as providing safer navigation within the virtual world [5]. Moreover, well-crafted virtualization allows for increased interactivity and personalization options, enriching the user’s experience and expanding the capabilities of VR applications among others, such as gaming and architecture [6].

Fundamentally, virtualization transcends its role as a mere technological requirement; it is paramount in shaping user perception and interaction within the virtual environment [7]. With the rapid advancement in technology and the enhanced resolution of VR headsets, there is a pressing need for virtualization to be of even higher quality. This progression not only demands virtualization techniques that yield authentic and reliable results but also insists that these methods become more accessible. They should evolve to be user-friendly, adopting automatic or semi-automatic functionalities to cater to a broader user base. As the technology becomes more widely available, the ease of implementing virtualization techniques becomes as crucial as their performance. This shift underscores the urgent demand for developing new frameworks and methodologies in this field. Such foundational advancements are necessary to support the creation of sophisticated high-level applications, ensuring that VR continues to grow in effectiveness and influence across various domains.

This is particularly true when considering the increasing significance of virtualization in indoor spaces [8], especially in today’s era where VR has been brought to the general public thanks to devices like the Oculus Rift (Meta Platforms, Inc., Menlo Park, CA, USA, https://www.meta.com/ (accessed on 30 May 2024)) and HTC Vive (HTC Corporation, Taoyuan, Taiwan, China, https://www.htc.com/ (accessed on 30 May 2024)) [3]. For many users, the primary interaction with VR occurs at their homes. This increase in domestic use highlights the need for sophisticated virtualization of indoor environments. As these locations become the main spaces for immersive experiences, tailoring and optimizing virtual representations of such areas becomes essential. This not only enhances the user experience but also ensures that VR applications become more immersive by being able to relate to and interact with familiar environments. This closer connection with users’ everyday surroundings helps to deepen the sense of presence and realism within the virtual realm. The evolution of VR devices and their increasing accessibility has thus placed an even greater emphasis on improving the virtualization of indoor spaces.

Regarding the main process of virtualization of indoor scenes, 3D reconstruction is a crucial component [9]. It involves creating a digital representation of an indoor environment, aiming to faithfully reproduce its geometry and features. This digital representation can take various forms, such as polygonal meshes or point clouds, either with color or without, derived from the collection and analysis of visual data (e.g., images, videos, or depth sensor information) collected from indoor settings. Beyond 3D reconstruction [10], virtualization also includes scene understanding, which detects objects [11], classifies them, and segments the instances in the scene [12] (for example, the individual chairs in a living room), or a combination of the three [4], allowing for customization in VR settings. It is typically executed after acquiring the digital representation of the environment to be able to manipulate, change, and interact with the elements in the virtual reality scenario, thus enhancing the user’s interactive experience. Moreover, to maintain a clean and efficient digital representation, these elements are often replaced by the most similar CAD models [3] in terms of shape positioned in the same location, orientation, and scale within the scene. Finally, another step of scene understanding is the layout extraction [2], referring to the estimation of the 3D planes that limit the scene (such as the walls and floor of a living room or bedroom). This leads to an improvement in the safety of the virtual scene, enabling not only a more accurate and efficient representation of the space’s limits but also safer interaction and navigation within it.

As previously mentioned, virtualization frameworks have significantly advanced; however, many of the current methods do not perform the complete process, resulting in a partial virtualization of the scene [4,13]. Additionally, there are persistent challenges that limit their effectiveness and applicability in VR settings [3]. These challenges span various domains according to the techniques applied, from automation to the quality and fidelity of the rendered environments.

Traditionally, 3D scene virtualization had been achieved manually [2]. Specifically, a team of graphic designers had to create every object in the scene and insert it into the scene in the correct place. Therefore, the scene had to be previously measured in detail. This method of manual design results in long, labor-intensive tasks. Moreover, if the application requires high-quality models, it increases the cost, thus making it unfeasible for smaller projects or companies. Additionally, hand-crafted models often lack the level of detail or accuracy required for certain applications, like architectural simulations. These limitations regarding high time costs and quality were partially solved by the advances in CV and DL techniques, which allowed some semi-automatic scene virtualization methods to emerge [4,14,15], but these methods still suffer from various limitations.

These restraints include the lack of complete frameworks that execute different steps in order to make the virtual scene fully interactive, realistic, immersive, and safe to explore. For instance, some methods lack a comprehensive 3D understanding of the scene and are focused primarily on the 3D reconstruction aspect, often overlooking the contents of the scene [15]. Furthermore, some approaches have limited precision, personalization, and interaction since they are focused only on safety navigation [5,16].

Another major limitation of current methodologies lies in the degree of automation within the frameworks. As pointed out by [4], many of these frameworks are not entirely automated, necessitating manual involvement at certain stages. This necessity often arises from the divergent methodologies employed at different phases of the process, which complicates the integration of these stages [3]. Consequently, substantial effort is required to achieve a cohesive and unified virtualization process.

In summary, the main challenges that virtualization frameworks face nowadays are the lack of a complete pipeline that executes all the different stages and the lack of completely automated methods that automatically generate the VR scene from the gathered images. We aim to solve these issues by introducing the Virtual Experience Toolkit (VET) framework. VET is designed to address the aforementioned limitations, offering a complete application that includes both the 3D reconstruction of the scene and the scene understanding step, ensuring a faithful representation of the layout and components of the scene, and a fully automated virtualization process that generates a customizable and interactive VR scene without intervention. Additionally, it is encapsulated in a user-friendly and intuitive Unity application that guides the user through the acquisition of the images, shows the processing stage, and loads the generated scene into a VR application for its exploration or customization. Concretely, VET performs a 3D reconstruction using an RGB-D camera and a dense-SLAM-based method, then applies a 3D scene understanding step composed by a layout estimation algorithm and a CAD substitution for the objects in the scene that also includes an automated version of the ScanNotate  [17] method. Finally, the information obtained is integrated into a digital scene, where the user can interact and edit the scene according to the necessity, using a custom and intuitive GUI. Moreover, the proposed solution is verified by our own scene dataset (consisting of various scenes such as bathrooms, bedrooms, conference rooms, and offices, among others) and ScanNet [18] to prove its effectiveness and precision. Herein, we outline the principal contributions of our work:

- Fully automated and user-friendly: The VET system stands out for its automation and ease of use. Upon acquiring the 3D reconstruction, it seamlessly virtualizes the captured data through an intuitive framework that guides the user. This integration is accomplished within a singular graphical application that harnesses the power of C++, Python, and Unity3D (Unity Software Inc., San Francisco, CA, USA, https://unity.com/ (accessed on 30 May 2024)).
- Complete virtualization: VET implements all the necessary stages to ensure a complete virtualization, including a 3D reconstruction to obtain a 3D replica of the scene and a scene understanding stage to process said scene. The scene understanding step is divided into two sub-stages—layout generation, responsible for generating the layout of the room and ensuring safety in the navigation, and the room objects substitution, responsible for detecting and substituting the objects for CAD models to heighten the interactivity and immersion.
- High quality: The VET framework produces high-quality results by implementing methods that have proven to be highly effective, such as the real-time 3D reconstruction method BundleFusion [19]; the current state-of-the-art (SOA) algorithms in the following pipeline steps, such as Mask3D [12], for 3D instance segmentation using the ScanNet  [18] and ScanNet200 [20] datasets; or an improved version of ScanNotate [17] for CAD retrieval and pose estimation.
- Personalization: The solution offers extensive customization options for users to accommodate their virtual experiences through a custom GUI.
- New layout method: VET presents a new, fast, and robust method for layout calculation of indoor rooms, employing the Robust Statistics-based Plane Detection (RSPD) algorithm [21] and the O-CNN [22] segmentation technique.
- Broad applicability: Our framework can be applied across a wide range of indoor scenes, working with an extensive array of classes, specifically 200 ScanNet object classes. This is demonstrated using a dataset acquired through VET that complements ScanNet, comprising RGB-D images, camera pose information, 3D reconstructed scenes, 3D scene understanding results, and complete virtualization.

By detailing these aims and goals, the paper will offer a comprehensive solution to existing challenges in the virtualization domain for VR, providing both theoretical and practical contributions to the field.

This paper expands the work of our previous research presented at the conference MetroXRAINE in 2023 with the following title: *Virtual Experience Toolkit: Enhancing 3D Scene Virtualization from Real Environments through Computer Vision and Deep Learning Techniques* [23]. While the conference paper provides an initial exploration of our framework, this paper offers a more comprehensive analysis and a more in-depth explanation of the methods, emphasizing the layout algorithm, the upgrades to the ScanNotate method, and the integration of the whole framework in a single application.

The remainder of this paper is organized as follows: Section 2 briefly reviews existing 3D scene virtualization methods according to the different stages performed. Section 3 presents the proposed framework, deeply explaining the different methods incorporated to obtain the complete 3D virtualization. Section 4 introduces the results obtained step by step and the discussion about them, along with a brief comparison with other known methods. Finally, Section 5 includes the overall conclusions and some suggestions for future work.

## 2. State of the Art

In recent years, 3D scene virtualization has gained widespread attention due to the growth in the usage of VR and increased automation, specifically in indoor scenarios [4]. Therefore, several solutions have been proposed for 3D realistic, personalized, immersive, and interactive scene generation, with varying approaches depending on the techniques used. Concretely, 3D reconstruction, scene understanding CAD retrieval, and pose estimation steps are required to link the physical and digital worlds in a virtual 3D scene while ensuring that the virtual scene closely mirrors the real world. Early 3D virtualization methods were performed manually. However, this solution is time-consuming and requires highly detailed information about the scene. Nowadays, the different virtualization methods vary depending on the methods used to perform the three main steps to carry out the virtualization process.

### 2.1. 3D Reconstruction

In the particular case of 3D reconstruction, one of the first methods to reconstruct 3D scenes automatically was photogrammetry. This traditional CV technique [24] is used to create 3D maps or models of objects or scenes, among others, from a set of 2D images using the information of the matching between features extracted from the 2D images. Despite the high volume of methods that use photogrammetry for 3D modeling, it is sensitive to image quality; challenging in featureless scenes and occlusions; and it also limited when texture-less elements, repetitive patterns, and reflecting surfaces are present in the scene like walls (in indoor scenarios). It is also computationally expensive [25]. Moreover, the main problem of using standard RGB images is the small field-of-view of 2D cameras [9] that can make contextual information insufficient for reliable full-room reconstruction. Another investigation field that overcome the mentioned drawback related to the field-of-view is the usage of full-view panorama images for indoor capture and 3D reconstruction [26,27]. Using 360-degree cameras for indoor 3D reconstruction faces two significant limitations: limited depth information and inconsistent resolution. These cameras can capture extensive views but struggle with accurately gauging depth, which is crucial for rendering precise 3D layouts and dimensions. Additionally, the panoramic images may have an uneven resolution, causing distant objects to appear less detailed or blurry. Together, these issues complicate achieving accurate and detailed 3D models of indoor spaces. Currently, with the introduction of accessible RGB-D cameras like Kinect (Microsoft Corporation, Redmond, WA, USA, https://www.microsoft.com/ (accessed on 30 May 2024)), different methods have been introduced to reconstruct 3D scenes from single or multiple RGB-D images based on dense SLAM. For instance, KinectFusion [28] creates a high-quality 3D model from the depth data of a Kinect camera. However, this method is constrained by the depth range of the Kinect sensor; thus, it only works for small, static scenarios. Since the introduction of KinectFusion, other algorithms based on dense-SLAM have appeared like InfiniTAM [29] and ElasticFusion [30], but all of them are computationally expensive. More recently, BundleFusion [19] takes advantage of SLAM combined with volumetric fusion to create detailed 3D models of large environments in real-time. Finally, one of the last novelties in this field is the introduction of Neural Radiance Fields (NeRF), a simple, fully connected network trained to reconstruct 3D scenes from a set of input images [31,32]. Currently, this approach is being applied in many practical applications such as robotic navigation and VR. Nevertheless, NeRFs suffer from large optimization times and slow rendering, especially for large-scale scenes.

Considering the aforementioned methods, in the literature, there are several 3D virtualization methods that use these methods to carry out the 3D reconstruction step. For instance, the Snap2Cad [10] solution is a 3D virtualization framework that uses a single RGB smartphone camera to reconstruct the 3D scenario, extracting planes and oriented bounding boxes information to finally replace the objects with 3D models. In addition, Lou et al. [13] presented a virtualization framework that uses as input a panorama image, detects 2D objects, estimates the layout, and finally applies a CAD retrieval algorithm comparing the CADs on a photo with the input image. On the other hand, Shapira et al. [14] presented a virtualization solution that uses KinectFusion data to reconstruct the 3D scene to be virtualized.

### 2.2. 3D Indoor Scene Understanding

Once the 3D reconstruction is obtained, it is required to interpret the scene content. In other words, different information should be estimated such as the spatial layout and the 3D objects that compose the scenario. In the specific case of layout estimation, it is commonly used to guarantee safe navigation in indoor scenarios. Traditionally, it was achieved using bottom-up image features such as local color, texture, or edges and the computation of the vanishing points considered geometric features. For example, Hedau et al. [33] introduced an iterative method that obtains the vanishing points and sample pairs of rays from those vanishing points to obtain the box parameters and, finally, it will extract the layout information. However, the results obtained by this method depend on the quality of the extracted features—in other words, it is sensitive to noise and occlusions. Due to the advances in RGB-D sensors, novel methods of layout estimation have appeared. Particularly, those methods are based on plane detection and plane intersection. Recently, layout estimation methods based on DL techniques have appeared. For instance, Dasgupta et al. [34] introduced a method that uses an FCNN that learns semantic surface labels with an optimization framework to generate the layout estimation. Moreover, Lee et al. [35] presented RoomNet, an End-to-End room layout estimation that extracts keypoints and connects them to obtain the final layout. The main drawback is the difficulty of finding suitable training data with enough layout information, because the performance is totally dependent on these data. Transferring this information into the field of 3D virtualization, it is important to mention that, in the literature, there are some methods that do not carry out this step. This is particularly observed in virtualization frameworks for outdoor scenarios like TransformMR [11], which is a 3D virtualization framework for outdoor scenes, where there is no limit. In the specific case of indoor scenarios, there are methods that do not ensure the safety navigation concept, such as the VRFromX method [4] and the method proposed by Han et al. [25], which do not estimate the layout to carry out the virtualization. On the other hand, the ScanToVR [3] framework uses RANSAC to detect the planes; then, it checks if these planes are composed by a minimum number of points to finally select those planes to compose the layout. However, this method depends on the planes detected by RANSAC and is also sensitive to occlusion. In addition, another virtualization framework that estimates the layout was presented by Luo et al. [13]. This solution uses LayoutNet [36], an algorithm that predicts the layout of the scene using a single panorama image as input. The main limitation of this method is the distortion of the performance produced by the usage of panorama images.

### 2.3. Instance Segmentation

Another step of the interpretation of the content in the scene is the processing of the objects present in the scene, such as the furniture of an indoor scene. This process aims to provide information about the 3D objects in the scene, which then can be used to define obstacles and substitute the reconstruction with more detailed versions of the objects. In this step, either a simple detection, classification, or segmentation of the objects can be carried out, or a combination of the three methods. Traditionally, this step was executed via clusterization methods; however, the advances in CV and DL have enabled the development of 2D and 3D methods based on diverse neural network architectures [37].

These CV- and DL-based methods have become the preferred technology for this step in the recent virtualization frameworks. The aforementioned methods can be classified based on the type of data they use to segment the scene; some frameworks implement a segmentation and classification (usually referred to as instance segmentation) step based on the RGB images used for the reconstruction, such as Snap2cad [10], which implements a convolutional neural network (CNN) with HRNet-W48 architecture for the segmentation. Another example is the work of Luo et al. [13], which similarly uses a Faster-RCNN network to detect furniture in panorama images. Given the origin of the data being RGB images, this approach presents some limitations, such as possible occlusions between the objects or the need to translate the 2D segmented information to the 3D space, which often generates noise and requires a refinement step for the 3D segmented object, such as that implemented in Snap2cad [10]. Due to the growth in popularity and availability of RGB-D cameras for indoor virtualization, some methods have emerged that combine the depth information with the RGB image to perform the segmentation. For example, Han et al. [25] fused the panoptic segmentation of the RGB image with the depth segmentation of the depth image, combined with an extra Euclidean clustering to improve the segmentation of the RGB image (i.e., two close chairs detected as one). Even with the improvement, the need to translate the 2D information to the 3D space still persists. To assess this limitation, other frameworks implement methods of 3D segmentation that are applied directly to the 3D reconstruction of the scene. A basic implementation of the aforementioned technology can be seen in the work of Moro et al. [38], where they apply a clusterization method to detect the objects in the scene that are not planes (walls, floor, and ceiling); however, the detected clusters of objects are used as obstacles to avoid, hindering the realism and interaction of the virtual scene. For a more detailed detection of the objects, ScanToVR [3] implements a 3D instance segmentation method based on 3D CNN, providing information about the class (chair, table, cabinet, etc.) and individually segmenting the different objects. Nonetheless, it is only able to detect eight classes, limiting the scalability of the framework.

### 2.4. CAD Retrieval and Pose Estimation

Once the information in the scene is interpreted, to increase the interactivity, it is required to individualize each 3D object in the scene. Huan et al. [15] introduced a method in the virtualization framework that isolates clusters; then, a mesh is reconstructed for each cluster. However, it is sensitive to noise in the scene, like points that belong to other objects and where the polygonal load is high. To reduce it, different methods based on replacing the 3D reconstructed object with the most similar CAD models appeared. For instance, the Snap2Cad framework [10] uses a VGG-19 CNN to obtain the CAD model retrieval and PoseFromShape method [39] to obtain the pose estimation of the bounding box. Despite the computation of the pose estimation, it is less accurate due to the background on the RGB image used to compute the pose estimation. Another framework is ScanToVR [3], which uses a model retrieval algorithm based on 3D CNN and then uses another algorithm to estimate the seven-DoF pose. Additionally, the pose estimation step works under the assumption of having all the objects in the scene placed upright, considering only one rotation on the Z-axis. Moreover, the usage of two different methods and the need to link both of them increase the computational cost. This same problem is also presented in the VRFromX [4] virtualization framework, where a model retrieval algorithm that uses the information of an ROI is used and the PointNetLK [40] method is responsible for the pose estimation step. Finally, Luo et al. [13] presented a framework that uses a Faster-RCNN network to carry out the CAD model selection. However, this framework uses 2D information instead of 3D to carry out this last step, thus losing the depth information.

### 2.5. Texture Synthesis

One extra step that makes the final scene more realistic and immersive is the texture synthesis of the planes of the room. Even if the generated room highly resembles the original space in its geometry and the objects that are present, the resemblance of the walls and floor plays a great role in the realism of the scene. Originally, the approach was to copy a crop of the desired texture in a tiled fashion for the walls; however, this results in undesirable regular paving [41]. In the last decades, texture synthesis methods have evolved to generate high-quality textures from sample images; nevertheless, we find few applications in virtualization applications. For example, OpenRooms [42] implements a texturing step but is not guided by the reference images; rather, it focuses on the photorealism and semantics of the scene. Another approach can be seen in PhotoScene [43] and PSDR-Room [44], which use a differentiable rendering to modify a base texture to match a reference image; however, these approaches require the manual selection of the initial material and make use of proprietary software. Additionally, another generalized method for texture synthesis is the use of CNN to generate a new texture from the reference image, such as the work of Li et al. [45], although these methods still face some issues for implementation in virtualization frameworks, such as the selection of the crops and the seamless tilling of the generated texture.

### 2.6. Summary

Following this review of the 3D virtualization literature, we summarize the methods in Table 1 in order to compare them to the VET framework. In said table, we can visualize that some of the methods are not complete and lack key steps to make a complete framework for the user, and some methods are not automated and require user input to generate the final VR scene. Some existing methods are both complete and automatic; however, they present some limitations. RealitySkins [14] is limited to four classes of objects, and the CAD placement is aligned to minimize the loss calculated with the occupancy grid of the original scene, meaning that the objects are a set of preselected CADs and are placed to fill the space that the original objects occupy, instead of substituting each independent object for a similar CAD model, hindering the realism and immersion of the final scene. The remaining method, GeoRec [15], uses the RGB images both for the layout estimation and the 3D object detection and substitution, facing issues such as occlusion and incomplete information and difficulty translating the 2D information of the images to a 3D scenario. In contrast, we present a framework that works with up to 200 different classes, prioritizes using the 3D reconstruction as the input data for the various steps, and substitutes the individual objects with the same class CAD models. Additionally, VET is fully automatic and incorporates every step of the workflow to ensure a high-quality virtualization, including the acquisition of the data; a dedicated step to generate a similar texture for the planes, which enhances immersion; and the VR environment, which is also customizable to the user demands.

## 3. Method

**Figure 1:**
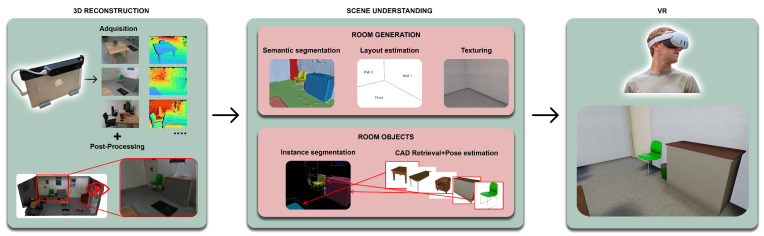
Pipeline followed by VET framework.

In this section, we provide a detailed explanation of the different stages that our proposed framework, VET, carries out to achieve 3D virtualization of real indoor scenes. Each step is applied to obtain a realistic, interactive, and safe environment that is geometrically and color-similar to the real indoor scene. The complete pipeline is carried out through different parts, as depicted in Figure 1, and is fully encapsulated in a Unity3D application that guides the user through the whole process. Furthermore, the generated room can be customized to meet the user’s needs using a user-friendly custom Unity3D Graphical User Interface (GUI). The postprocessing and the 3D reconstruction, along with the scene understanding steps, are processed in a PC with specifications that can be found at the end of Section 4, and the VR step—where the user can visualize, interact, and edit the scene—is loaded onto a VR headset. It is important to remark on the significant efforts made to guarantee seamless integration and compatibility across diverse algorithms and methods, spanning from the initial acquisition phase to the ultimate virtualization stage. This meticulous process is conducted in a manner that remains transparent to the user, ensuring a smooth and intuitive experience.

### 3.1. 3D Reconstruction

3D reconstruction is the foundational step in our Virtual Environment Toolkit (VET) for indoor space virtualization, employing RGB-D imagery to accurately recreate real-world environments in a three-dimensional context.

The core of our methodology is BundleFusion, an advanced technique that originates from the domain of Dense Simultaneous Localization and Mapping (SLAM). Proposed by Dai et al. [19], BundleFusion has established itself as a robust solution for generating real-time, high-fidelity 3D models of interior settings. The technology shines in its comprehensive treatment of volumetric data, marked by a multi-tiered approach that commences with meticulous frame-to-model camera tracking. This initial phase harnesses a fine-grained iterative closest point (ICP) algorithm, ensuring acute preservation of detail in real-time.

BundleFusion’s robustness is anchored in its global pose graph optimization, which meticulously refines camera trajectories to minimize drift and bolster the trajectory estimation process. A salient feature of this technology is its dense surfel-based fusion, paired with a loop closure detection mechanism, ensuring the structural coherence of the model by rectifying cumulative drifts through pose graph optimization.

A globally consistent surface reconstruction complements this process by unifying and refining surfels, culminating in a seamlessly integrated global model. BundleFusion stands out by implementing an energy minimization strategy that effectuates simultaneous refinement of camera poses and geometry, enabled by a global non-rigid deformation framework, thus ensuring fidelity to sensor-captured data.

Addressing the challenges posed by large-scale environments, BundleFusion effectively manages both memory and computational demands with an efficient hierarchical data structure. This enables incremental integration of depth frames into a comprehensive model without compromising on detail—a significant stride beyond conventional SLAM systems.

The integration of BundleFusion within VET enhances the precision of our 3D reconstructions. Also, it aids the user during the reconstruction process by providing real-time feedback on scanned and unscanned areas. To further augment this precision, we incorporated a more accurate volumetric fusion algorithm, as developed by Dong et al. [46], providing VET with the ability to produce superior-quality models compared to those generated solely by BundleFusion in a non-real-time reconstruction that typically requires an average of 4 min to perform the 3D reconstruction.

VET’s adaptability is evidenced by its support for an extensive range of RGB-D cameras, including popular models such as the Intel^®^ Realsense D435 and D415 (Intel ^®^ Corporation, Santa Clara, CA, USA, https://www.intel.com/ (accessed on 30 May 2024)); the Stereolabs ZED2i (Stereolabs Inc., San Francisco, CA, USA, https://www.stereolabs.com/ (accessed on 30 May 2024)) cameras, which were instrumental in our dataset compilation; and the Structure Sensor (Structure, Boulder, CO, USA, https://structure.io/ (accessed on 30 May 2024)) leveraged in the ScanNet initiative.

Postreconstruction refinement is crucial to optimize the 3D models for further processing. Applying Quadric Decimation [47] reduces polygon counts drastically, improving computational efficiency. We also mitigate sensor-induced artifacts by removing isolated noise clusters, an improvement informed by the work of Kadambi et al. [48]. The culminating step aligns the reconstructed scene with the ScanNet dataset’s coordinate system [18], facilitating seamless integration and comparability.

It is crucial to bring to attention the importance of the data acquiring step. The VET framework generates the virtualization based on the RGB-D images gathered; therefore, a high-quality acquisition is needed, taking the necessary time and reviewing the quality of the scanning through BundleFusion. Moreover, this method has some limitations during the acquisition: sudden or quick movements and reflective surfaces generate a temporary loss of the camera pose, effectively losing the affected frames, and translucent or transparent objects will not be correctly scanned.

VET uses an indoor scene understanding approach to build on the reconstructed framework. This dual process comprising semantic segmentation and layout estimation, alongside instance segmentation and CAD model retrieval and alignment, is conducted in parallel. Such comprehensive processing paves the way for accurate digital scene virtualization, reflecting the intricacies and nuances of the physical space.

### 3.2. Semantic Segmentation and Layout Estimation

The layout computation begins with the semantic segmentation of the scene, aiming to label the reconstructed 3D environment by dividing it into semantically distinct regions corresponding to indoor objects, such as furniture, windows, or doors, and room parts like the walls and floor. For this task, we employ O-CNN (Octree-based Convolutional Neural Networks) [22], which has demonstrated high efficiency and precision within the ScanNet dataset.

Particularly, the approach uses octree representation to efficiently detect the features of the point cloud and the 3D shapes present in it, calculating average normal vectors from finely sampled leaf octants. This representation is then used to train a semantic segmentation CNN network showing improved performance over standard CNN for 3D data and tasks. For this step, VET specifically uses an octree-based U-net trained for the semantic segmentation of the ScanNet dataset, which provides a semantically segmented 3D scene that aligns with our requirements for the following stages. In other words, all the different objects are semantically segmented depending on the indoor object type. Specifically, we leverage ScanNet’s existing semantic classes, such as walls, floors, cabinets, doors, and windows, which are potential layout parts. The scene undergoes filtration to exclude all but the aforementioned classes, resulting in a streamlined point cloud for further analysis.

The layout estimation phase is crucial as it defines the spatial boundaries within a room by estimating 3D planes and corners, which are identified at the intersections of these planes. The method proposed follows Algorithm 1, where the variable points are the points that compose the 3D reconstruction and $color_{layout}$ refers to a list of those segmentation colors corresponding to layout selected labels. Also, target_points makes reference to the selected points from the mesh that will be used to estimate the layout. In addition, planes stores the estimated ones from which we will compute the normal ($n_{plane}$) and the center (c_pl) if it follows the Manhattan rules. Finally, the information required to obtain the layout (intersection points and planes) is stored in the layout variable.

Although RANSAC is widely recognized for plane detection [38,49], we adopt a novel approach known as Robust Statistics-based Plane Detection (RSPD) [21]. RSPD offers significant advantages, including faster computational performance, fewer initial constraints, and enhanced accuracy. From the semantic segmentation step, we extract the points segmented with the target labels and those that are part of the layout, and by employing RSPD, we detect planes within the point cloud. These planes are then processed based on their normal vectors to isolate the ones pertinent to the layout and also remove those duplicated planes.

Specifically, to detect duplicate planes, we consider both normal vectors and the centers of the planes. By merging this information, we can detect closely parallel planes. After studying typical features of indoor scenes, we decided to set an experimental distance threshold of approximately 10 cm, meaning that any pair of walls that are parallel and their centers are closer than the said threshold will be considered duplicate, and one of them would be eliminated since, typically, walls are not that close in indoor settings.

Once the filtered planes are estimated, the intersection between them is computed, obtaining the corners that will compose the layout assessment. Finally, the resultant selection of planes and corners forms the basis of our layout estimation.
**Algorithm 1** Algorithm for layout estimation**for** *p* **in** points **do**    $p_{color}\leftarrow get\text{\_}color\left(p\right)$    **if** $p_{color}$ **in** $color_{layout}$ **then**        target_points←p    **end if****end for**planes←estimate_planes(target_points)**for** pl **in** planes **do**    $n_{plane}\leftarrow get\text{\_}normal\left(pl\right)$    **if** $n_{plane}$ **in** manhattan_rules **then**        plane_select←pl        c_pl←get_center(pl)        **if** c_pl−c_planes<0.1 **then**           plane_select←remove(pl)        **end if**    **end if****end for**layout←intersection(plane_select)

Although this method has demonstrated its efficiency during the validation of VET, it still presents some limitations. The layout estimation step operates under the Manhattan World assumption, albeit with some flexibility. This means that while walls can deviate slightly from being parallel, the method is not suitable for scenes featuring diagonal or circular walls. Moreover, walls may not be detected if they are crystal walls, since the scanning method will not capture it; if they are partially scanned; or if they present some gaps.

### 3.3. 3D Instances Segmentation

To deal with each object independently, all the 3D objects in the scene should be segmented as independent instances. For this step, we adopt Mask3D, which currently stands as the state-of-the-art (SOA) for 3D semantic instance segmentation [12], particularly evidenced by its performance on ScanNet200 datasets. Using a pretrained model on ScanNet200 dataset, we automatically obtain the 3D instances segmented aligned to our requirements.

Concretely, Mask3D demonstrates proficiency in predicting semantic classes and instance features, such as the full semantic and geometric detail of each instance in a 3D scene. This capability makes Mask3D suitable for our segmentation tasks. The model combines a sparse convolutional feature backbone with Transformer decoders, enabling efficient whole-scene processing. As a result of this combination, Mask3D obviates the necessity for manual fine-tuning and the heuristic postprocessing that typically accompanies traditional methods.

Likewise, during the critical training phase, Mask3D uses bipartite graph matching to align predicted masks with the ground truth accurately. This method ensures a more precise and effective training process over simpler matching techniques.

Significantly, Mask3D has established new state-of-the-art benchmarks across various datasets, such as S3DIS [50], STPLS3D [51], ScanNet [18], and ScanNet200 [20]. These results further prove that Mask3D can yield high-quality segmentation results and can adapt to a higher number of classes.

Nonetheless, Mask3D presents some limitations in specific conditions. Mainly, in cases where the objects are very close, Mask3D may predict only one instance for more than one object. Further, in cases where the objects are partially scanned, the predictions may not be accurate.

The incorporation of Mask3D within our research not only aligns us with the current state-of-the-art but also allows us to take advantage of its advanced technological framework, enhancing our 3D instance segmentation process. The model’s inherent capacity to predict intricate queries based on scene semantics and geometry considerably minimizes the need for human intervention, yielding superior segmentation accuracy. To successfully implement the method in VET, we modified the data loading step to make it process a single point cloud and adapted the output to the following method’s input to incorporate it seamlessly into our workflow.

### 3.4. CAD Retrieval and Alignment

Once the instance segmentation is carried out, it is required to automatically and precisely replace the instances of the different objects in the 3D scene with CAD models in the same position, orientation, and scale as real-world objects. This process is performed to be able to modify the shape, appearance, and spatial location of the elements in the scene later using our GUI while reducing the polygonal load of the scene. To do so, VET incorporated the recent method proposed by Ainetter et al. [17] known as ScanNotate, which replaces the objects detected in the scene with semantically and geometrically similar CAD models. ScanNotate first estimates the 7 DoF pose of the objects; then, it retrieves the closest matching CAD by comparing the labeled object with the CAD models of the same class. In addition, this method joins objects of the same class into clusters to assign the same CAD to all the objects in one cluster if they are similar enough and, finally, it applies a refinement step to make the results more precise.

Regarding the dataset, the original implementation of ScanNotate works with the ShapeNet [52] dataset, a large-scale dataset designed for object recognition and understanding in computer vision and machine learning tasks. This dataset covers a wide range of object categories, including everyday items and furniture, among other least common classes. For its use in ScanNotate, multiple classes of this CAD model dataset are mapped to the ScanNet classes so the algorithm can search for the CAD models in the respective categories. Nevertheless, we made specific improvements to the dataset and its management. Specifically, we reduced the dataset by eliminating the outdoor classes and keeping only indoor objects, and filtered non-realistic and repeated models from the most populated classes: “chairs” and “tables”. Additionally, we created the refrigerator class isolating models from the “cabinet” category and complemented the dataset with models from ModelNet [53], creating these new classes: range hood, toilet, curtain, door, and sink. Finally, since VET uses ScanNet200 for instance segmentation, we mapped the 200 labels to the modified dataset, resulting in a more optimal and versatile dataset. This modified version of the dataset is available at https://github.com/Pamogar/VET-IndoorDataset (accessed on 30 May 2024).

However, ScanNotate presents two main limitations for our pipeline. First, the method is conceived as an annotation application. Therefore, it requires as an input a list of 3D bounding boxes of the objects and a list of the images where the objects are visible. Second, the processing time is proportional to the size of the dataset since it checks every model for the instance selected, thus making it highly time-consuming. To tackle these limitations, VET implements a preprocessing step that calculates the 3D bounding boxes from the instance segmentation results and selects the best images for each object, and a fast mode that implements the work of Beyer et al. [54] to preselect a reduced list of possible CAD models, significantly speeding the CAD retrieval and alignment step.

#### 3.4.1. Preprocessing

As mentioned above, the ScanNotate method requires a preprocessed input for the scenes. This input is mainly a list of 3D bounding boxes, a list of selected images for the objects, and the class of the object.

Starting with the 3D bounding boxes, using the results of the instance segmentation step, VET extracts the instance class and the 3D points that belong to the instance and computes a 3D bounding box aligned with the floor plane (only rotated in the height axis). In the case of some special classes, such as chairs and tables, the bounding box is extended down to the floor to ensure that it is correctly placed since, typically, these classes are always in contact with the floor, and the instance segmentation step may fail to include the legs in the cluster.

To refine the selection of object images, VET implements an initial filtering stage, addressing the common challenge of managing the extensive volume of RGB images. Given the prevalence of numerous, often indistinguishably similar images of any given object, it becomes crucial to streamline the selection process. To achieve this, our method incorporates a custom filtering algorithm. This algorithm systematically excludes images by analyzing the camera’s rotation and position, employing a sequential approach. This strategic filtering not only simplifies the dataset by removing redundant or less informative images but also ensures the selection of the most representative and diverse images for further analysis. This filter implementation is depicted in Algorithm 2, where $pos_{ref}$ and $rot_{ref}$ are the translation and rotation of the camera for a reference image, $img_{i}$ is a given image in the image list, $pos_{ref}$ and $rot_{ref}$ are the translation and rotation of the camera for the given image, $pos_{diff}$ and $rot_{diff}$ are the difference in translation and rotation between two camera poses, and list_filtered is the list of filtered images.

Starting with the first image, we use the pose of the camera as the reference, read the following image poses, and compare the poses until one image exceeds the threshold of minimum rotation or translation, which can be configured. Then, we add this image to the filtered list and use its pose as the reference to decide the next images. Iterating over all the RGB images obtained in the scan, we retrieve a list of filtered images, ensuring that the images are sufficiently different.
**Algorithm 2** Algorithm for filtering the images$pos_{ref},rot_{ref}\leftarrow get\text{\_}pose\left(img_{0}\right)$**for** $img_{i}$ **in** img_list **do**    $pos_{i},rot_{i}\leftarrow get\text{\_}pose\left(img_{i}\right)$    $pos_{diff}\leftarrow abs\left(pos_{ref}-pos_{i}\right)$    $rot_{diff}\leftarrow abs\left(rot_{ref}-rot_{i}\right)$    **if** $pos_{diff}\geq pos_{th}$ **or** $rot_{diff}\geq rot_{th}$ **then**        $list\text{\_}filtered\leftarrow img_{i}$        $pos_{ref},rot_{ref}\leftarrow get\text{\_}pose\left(img_{i}\right)$    **end if****end for**

At this point, for every instance, we sample the 3D points of the instance following a voxelization method and use the camera intrinsics and the pose of the images to project the 3D points into the image. Additionally, we compare the depth of the 3D points and the depth image to determine if the point is visible in the image or is occluded.

Next, we annotate the number of visible points of the object for each image and store the value; once we have processed all of the images, we can calculate the score of the images following Equation (1), where inst refers to a given instance of one of the objects detected in the scene, *i* refers to a given image in the list, $vis_{inst,i}$ is the number of points in an image *i* for the instance inst, and $max_{i^{\prime }}\left(vis_{inst,i^{\prime }}\right)$ is the maximum number of points visible in a single image for the instance inst.

(1) $$score_{inst,i}=\frac{vis_{inst,i}}{max_{i^{\prime }}\left(vis_{inst,i^{\prime }}\right)}$$

Therefore, to calculate the score of each image, we first find the maximum number of visible points for a particular object across all the images. Then, for each image, we divide the number of visible points for that object in that image by the previously found maximum. This gives us a score that represents the proportion of visible points in that image compared to the maximum number of visible points across all images. This results in a list of scores of images, ranging between 1 and 0, where the image or images with a score of 1 are the images where the object is most visible, and the images with a 0 do not contain the object. In summary, the closer the score is to 1, the more visible the object is in the image, and the closer it is to 0, the less visible it is. From this score list, VET extracts the top ten images with the higher scores, to use them in the CAD retrieval and alignment step. Additionally, given that the algorithm can also work with fewer images, VET filters out images with a score lower than 0.05 to ensure that the instance is clearly visible in the selected images.

This process ensures a high-quality image selection, with high visibility of the object and varied angles. Additionally, it enables the ScanNotate method to be used in an automatic setting, solving one of the main limitations observed.

#### 3.4.2. Fast Mode

As previously disclosed, one of the limitations we found with ScanNotate is the processing time with the ShapeNet dataset, especially with classes linked to the chairs and tables. To solve this issue, we opted for implementing a different CAD retrieval method previous to the ScanNotate method; with this approach, we preselect a reduced number of CAD models for classes that are types of chairs and tables and feed it to ScanNotate to obtain the most similar CAD and the alignment.

To select the reduced CAD model dataset, VET implements the work of [54]. This approach starts with the calculation of a 32 × 32 occupancy grid of the CAD datasets and the 3D instances; then, the grids are encoded using a 3D convolutional neural network with residual connections that produce a 128-dimensional embedding. Finally, the embeddings are compared, calculating the pairwise cosine similarity loss, and the CAD models with the lower value are selected as the most similar.

The first step for implementing this method is the preprocessing of the dataset. In order to eliminate the need to encode all the possible CAD models in every execution of VET, we preprocess the whole dataset offline, saving the results into a file that VET can then load when required, reducing the computational cost of this step. This file is also available at the dataset github.

For the implementation in the VET pipeline, once the 3D points of the instance and the 3D bounding box have been extracted, the instance is transformed into a 32 by 32 occupancy grid. However, given that the bounding box is not oriented, we create four copies of the instance and rotate it by 90 degrees on the Y axis (height in PyTorch3D coordinate system). At this point, the four versions of the instance are processed into the 128-dimensional embeddings and are compared with the CAD dataset via cosine similarity. This produces a loss matrix from which VET extracts the top k most similar CAD models from the four iterations of the instances, with k being a configurable parameter set by default at 50. Finally, the resulting list is filtered to ensure that the models are not repeated, and the corrected list is fed into the ScanNotate algorithm, overriding the base dataset and using this reduced one instead.

With this implementation, VET is able to reduce the computational cost of the CAD retrieval and alignment step while retaining high-quality CAD retrieval results and making use of the efficient ScanNotate method for the orientation of the selected CAD model. This step generates a list of the CAD models that better resemble the original objects they are substituting; have the correct position, orientation, and scale; and are prepared to be fully interactive in the VR scene.

### 3.5. Plane Textures

Once VET obtains a fully virtualized version of the original room, with interactive and lightweight furniture and defined limits for the scene, we add an extra step to improve the realism and immersion of the whole experience, specifically by adding texture to the floor and wall planes, using the original room textures for reference.

For this step, we opted for a texture synthesis approach that generates a new texture base on an input crop of the planes. Precisely, we extract the crops of the planes from the images taken for the 3D reconstruction of the scene. These crops then serve as inputs to the isolated texture generation module of Plan2Scene [55]. This module stands out for its adeptness at synthesizing a diverse range of textures. Remarkably, it achieves this without necessitating pretraining specific to the crop at hand. The result is a synthesized texture for the planes that not only closely mirrors the original but also features meticulously corrected borders. This enhancement ensures seamless integration, effectively eliminating any conspicuous seams and promoting a pristine, cohesive appearance throughout the scene.

For the crop selection part, we use a similar approach to the preprocessing of the CAD retrieval and alignment step to select the images. We obtain a list of 3D points for each wall using the semantic segmentation and layout results. Then, using a voxelization method, we sample points from each wall, project said points to the RGB images, and select the image where the highest quantity of points are visible. For the next step, using Grounded SAM [56] with the prompt “wall” or “floor” (according to the type of plane), we obtain the segmentation mask that indicates where the planes are in the image. Next, we extract five rectified crops of the masked area using the paired depth image.

At this point, we feed the rectified crops into the texture generation module of Plan2Scene. This method first computes a texture embedding for each surface crop, then decodes a texture from said embeddings, and finally applies a postprocessing step to the crop to make it seamless. For the first two steps, this module implements a version of the approach of [57], modified to enhance the disentangling of the color, pattern, and substance type and to allow the use of a single model for various substances. This method is applied for all the crops of the planes, and the best crop for each instance is selected using the L2 difference of VGG Gram Matrices with the generated texture. This step ensures the selection of the best possible crop, avoiding the inclusion of artifacts and edges in the textures. Finally, the postprocessing step is carried out with the Embark Studios Texture Synthesis Library to correct the borders of the generated texture and ensure that the seams are not noticeable once the texture is tilled into the planes.

Additionally, in cases where the plane’s texture is just a plain color, VET assigns the median of the color of the 3D points assigned for each plane.

Regarding some limitations of this approach, lighting variations may affect the retrieved texture and low-quality RGB-D cameras may generate lower-quality texture, since the synthesis is heavily influenced by the input crop extracted from the RGB images acquired.

With this extra step, VET reaches a higher level of realism and immersion to further enhance the user experience and the perception that the virtual scene is equivalent to the real one in terms of geometry and appearance.

### 3.6. Integration

**Figure 2:**
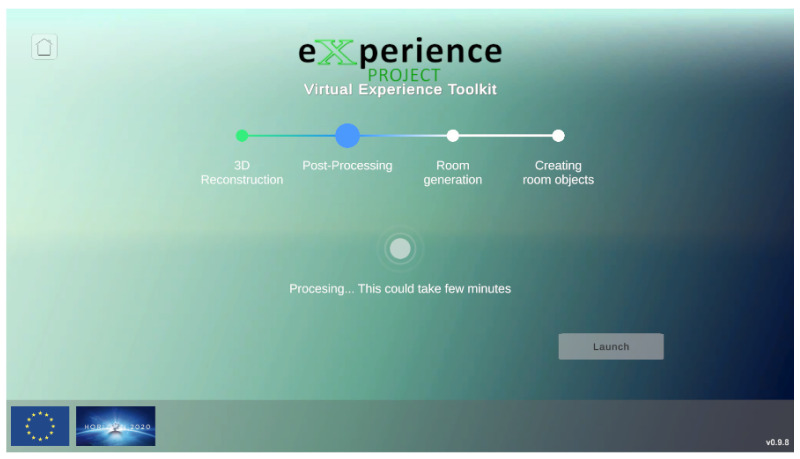
GUI created for the VET framework.

VET was developed using Unity3D (Version 2020.3.39f1). The application presents a custom GUI (Figure 2) to guide the user during the whole pipeline and to show the current state of the processing, showcasing the different steps of the pipeline and its progression.

At the same time, the Unity3D application is responsible for executing all the different C++ and Python processes configured to work automatically using a single configuration file in the background. These different processes are performed following the workflow of the diagram in Figure 1.

**Figure 3:**
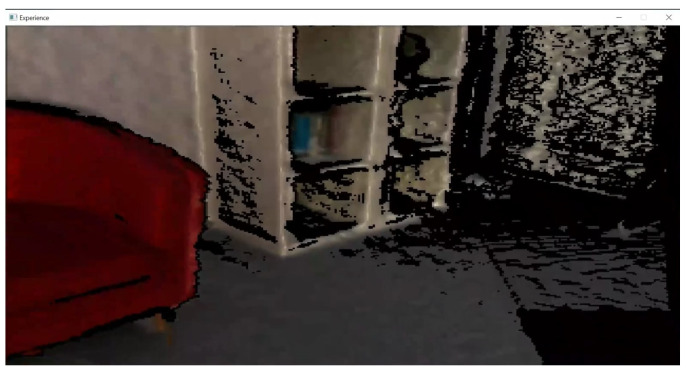
GUI of BundleFusion showing the reconstruction in real-time.

Starting from the 3D reconstruction, VET uses the BundleFusion method to scan and generate the 3D reconstruction of the fly. This method incorporates its own GUI, shown in Figure 3, from which the user can observe a preview of the 3D reconstructed scene in real-time. Once the 3D reconstruction is completed, VET carries out the postprocessing of the 3D scene and then executes two different stages in parallel.

The first stage handles room boundaries, starting with semantic segmentation, followed by layout estimation, and concluding with texture extraction for walls and floors. The second stage focuses on the room’s objects, utilizing instance segmentation to facilitate CAD model retrieval and object orientation. Executing these stages in parallel optimizes hardware use and minimizes processing time, ensuring a detailed and accurate reconstruction of physical spaces.

Finally, the generated 3D room is loaded into a VR application developed also with Unity3D, where the scene is displayed through a realistic render with illumination. Specifically, the illumination is added through a real-time point light from Unity, which generates realistic illumination that interacts with the uv maps of the models, which are proven to be of high quality. In this environment, the user can inspect the final result and fully explore the scene, moving freely both by walking and using teleportation, which allows VET to be used in any type of environmental setting.

**Figure 4:**
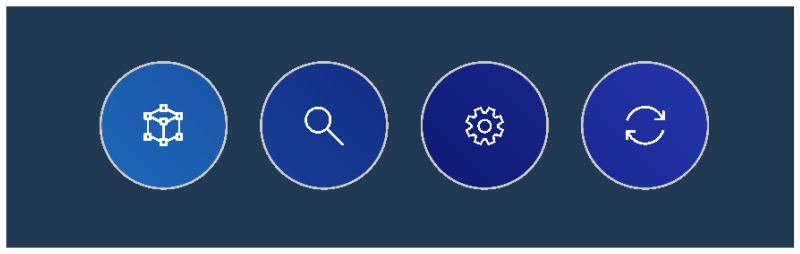
Edit option buttons from left to right: Edit rotation and scale, Change model, Edit colors, and Undo.

**Figure 5:**
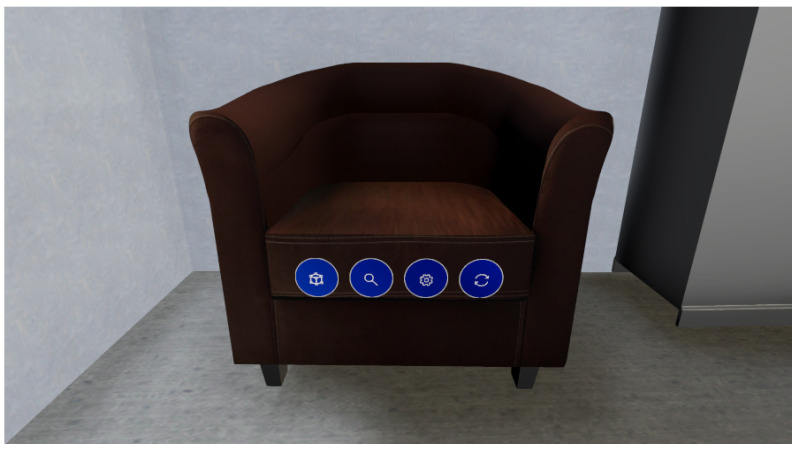
GUI for the editing of CAD models in the VR scene.

Additionally, VET allows the user to interact completely with the scene and all the objects present in it, including walls, doors, windows, furniture, etc. VET also offers a high level of customization, enabling the user to edit the objects in many ways. Figure 4 shows the options the user has to edit any placed object in the form of buttons with an icon indicating the option, and Figure 5 depicts how the user would see these buttons in the VR scenario.

**Figure 6:**
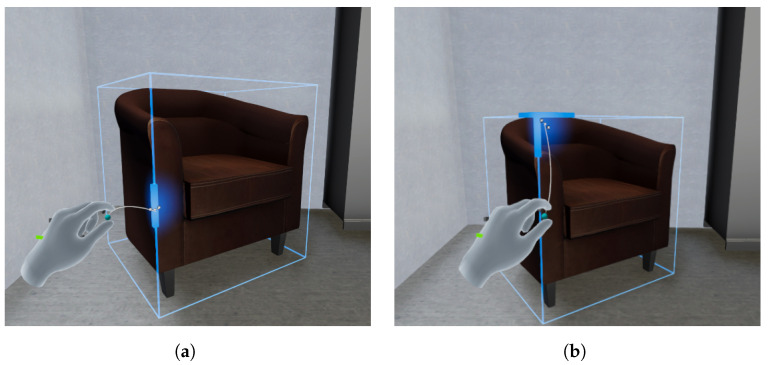
Examples of an edited object using the custom GUI of VET. (**a**) Translated and rotated object. (**b**) Scaled object.

In addition to these options, the user can simply select and grab the object in order to move it through the scene to a different location. Regarding the edit menu, the leftmost button allows the user to freely edit the rotation and scale of the model. Specifically, when selected, VET shows the 3D bounding box of the object; by grabbing the sides of the bounding box, the user is capable of rotating the object one axis at a time. Figure 6a shows a translation and rotation of an object placed automatically by VET. Moreover, grabbing the corners of the bounding box allows the user to uniformly scale the object to modify its size, as depicted in Figure 6b with the same object.

**Figure 7:**
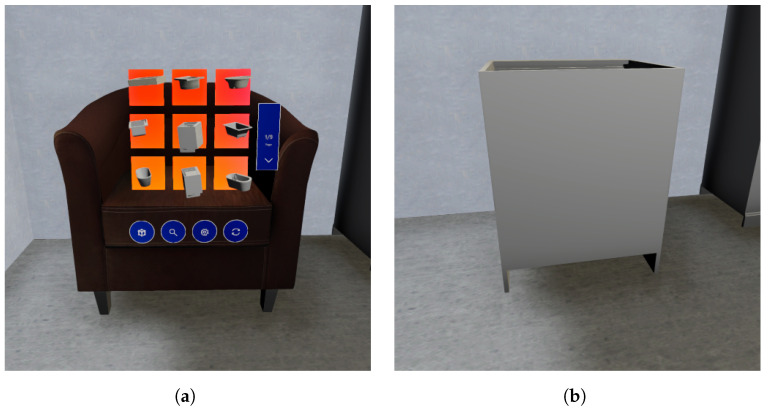
Example of a replacement of the CAD model of an object using the custom GUI of VET. (**a**) Custom GUI for the CAD replacement. (**b**) Replacement of the CAD model.

Additionally, VET enhances user interactivity by featuring an option to select an alternative 3D model for any given object. This functionality is accessible through the second button on the editing interface. By default, when activated, this feature presents a curated selection of 3D models from the same category as the object in question, enabling users to easily find a suitable replacement. However, for those seeking more creative freedom, this tool also offers the flexibility to explore and choose from models across different categories, thus broadening the scope for customization and innovation in the replacement process. Figure 7 shows an example of the change of the model of an object; specifically, Figure 7a displays the custom GUI of the Change model option, showing a preview of the CAD models in the selected class, along with options to show the next or previous CAD models or change it to another class. Finally, Figure 7b shows the object with a new model selected by the user.

The remaining options are “Edit colors”, where the user can change the colors of the CAD model selected, in order to adjust the appearance of the object freely, and an “Undo” button, which allows the user to reverse the changes made to the object up to the original configuration generated by VET; this enables the user to edit and interact with the scene without fear of messing up the scene since there is always an option to go back. Thanks to these capabilities, the range of applications for VET expands into tasks such as interior design and renovations.

Additionally, if there are objects detected that do not have a CAD model representation in our database, VET generates a green cube of the dimensions and orientation of the detected object, which serves as an initial anchor in case the user wants to select a CAD model from the existing database, load a custom CAD model to use for the representation, or load an image to use for the representation if the detected object is a picture. These objects can also be deleted from the scene if there is no available representation, so as to not hinder the immersion and realism of the scene.

In conclusion, the cohesive implementation of VET, developed using Unity 3D, creates a seamless and user-friendly experience, guiding the user throughout the entire pipeline and providing real-time feedback on the processing state. The final output, loaded into a VR application, allows users to effortlessly observe, navigate, and interact with the 3D room, with additional editing capabilities provided through a user-friendly interface, which helps compensate the limitations of the methods implemented and achieve a higher level of realism. Overall, the VET pipeline is easy to use, versatile, and fully automatic, offering a comprehensive solution for 3D scene generation and manipulation.

## 4. Results

We developed a specialized dataset from the Universitat Politècnica de València (UPV), featuring 30 unique indoor scenes for testing VET. This dataset, inclusive of necessary files for scene reconstruction and an extra demo room for detailed evaluation, is aimed at demonstrating our framework’s effectiveness. It is publicly available for research and further exploration at https://github.com/Pamogar/VET-IndoorDataset (accessed on 30 May 2024), serving as a resource for those interested in the field of 3D scene analysis.

**Figure 8:**
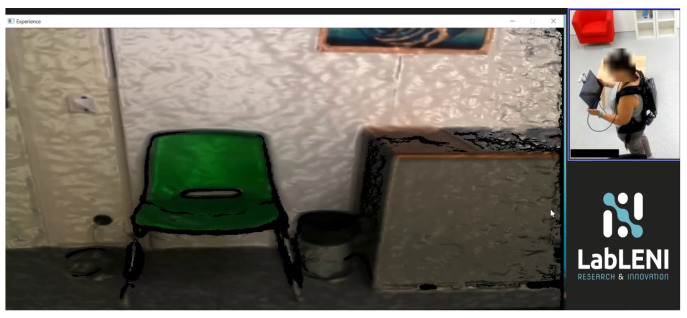
Real-time reconstruction of a scene using BundleFusion through VET.

**Figure 9:**
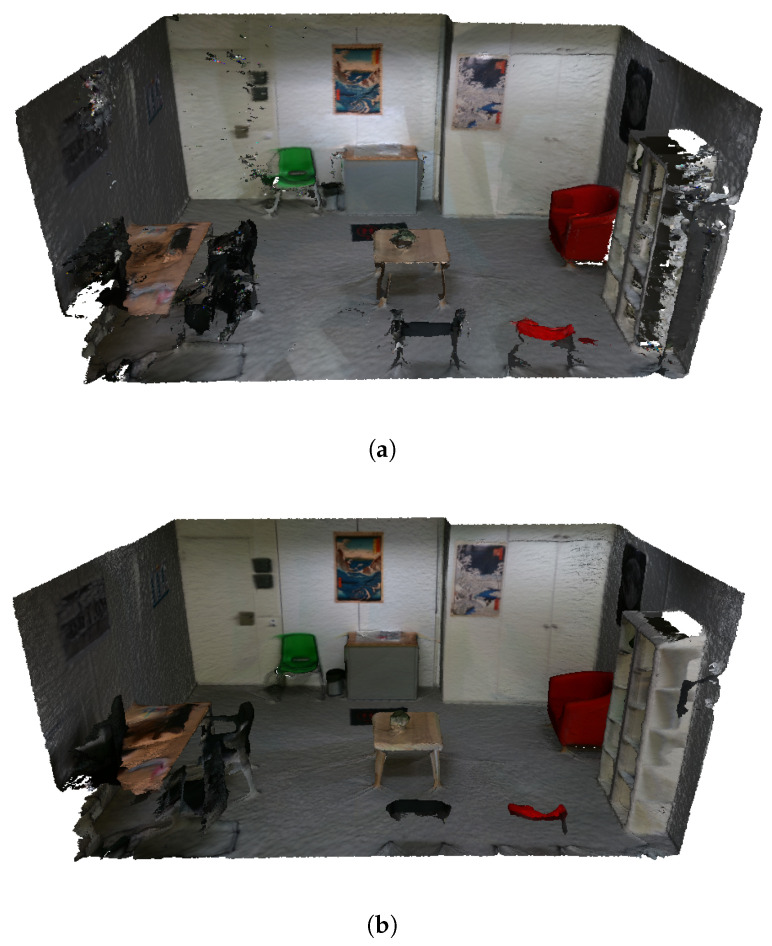
Reconstruction results. (**a**) Reconstruction of the scene using the BundleFusion method. (**b**) High-quality reconstruction of the scene using the method by Dong et al. [46].

In this section, to qualitatively evaluate our method, we present the results of all the independent stages, along with the final virtual scene in the VR application. The workflow starts with the scanning of the real room; here, the user is aided by the BundleFusion method, showing a real-time reconstruction of the scene, as shown in Figure 8. This helps the user recognize the parts that have already been scanned and the parts that still need to be scanned or may benefit from a re-scanning. Figure 9a shows a completed 3D reconstruction using the BundleFusion method implemented in VET.

**Figure 10:**
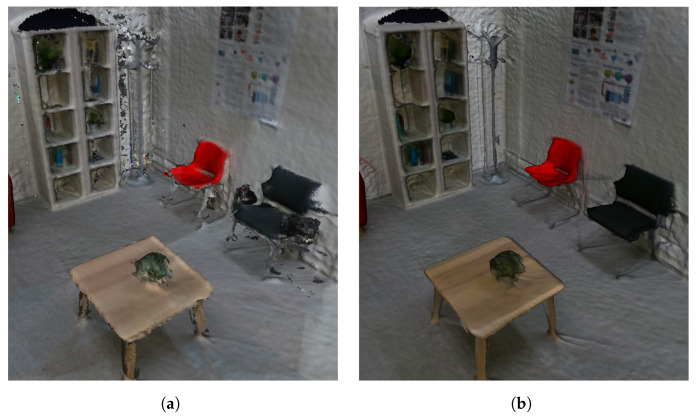
Side-by-side comparison of the reconstructions between the two methods implemented in VET. (**a**) BundleFusion reconstruction. (**b**) High-quality reconstruction.

Additionally, VET employs the method introduced by Dong et al. [46] to create a higher quality 3D reconstruction of the scene from the acquired data at the cost of the computational time of approximately 4 min. The same instance reconstructed with this alternative method can be seen in Figure 9b. Furthermore, to highlight the differences between the reconstructions and to compare them, Figure 9 shows both reconstructions and Figure 10 presents a side-by-side view of the results. In these images, we can observe that the reconstruction made with the method of Dong et al. [46] generates fewer artifacts and holes and an overall higher quality reconstruction, with more detail and smoother edges.

Once the 3D reconstruction is generated, VET launches the next two stages: the room limits extraction and the independent object substitution. Starting with the room limits, the first step is the semantic segmentation of the room, where we aim to divide the point cloud into different regions with labels to identify the layout components of the scene. Specifically, we use an octree-based u-net, trained following the method of O-CNN. In particular, this approach obtains an mIoU (mean Intersection over Union) of 0.762 on the ScanNet dataset—this metric calculates the overlap between the predicted and ground truth regions by measuring the intersection of their areas relative to their union, providing a reliable metric to assess the accuracy of segmentation models. This result places this method at the top of the SoA, notably performing better than Fully Convolutional Networks (FCN). Furthermore, this method presents high scores for the classes that relate to the layout–specifically, the wall, floor, cabinet, door, and window. The values are displayed in Table 2. Regarding computational cost, the processing times vary according to the size of the scene; for a typical room such as the one pictured in the previous figures, this method takes an approximate time of 50 s.

**Figure 11:**
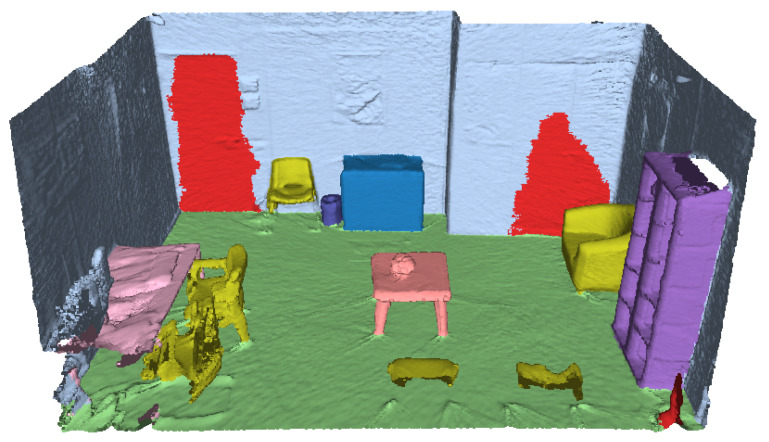
Result of the instance segmentation step of VET.

Applying this method to the 3D reconstruction of the scene, VET obtains a color-coded segmented scene, such as the one present in Figure 11. We can observe that the method correctly segments all the walls and the floor (light blue and green) and is capable of detecting objects close to them and segmenting them correctly, ensuring a high-quality segmentation of the elements that compose the layout of the scene, even if they are objects such as doors or windows.

**Figure 12:**
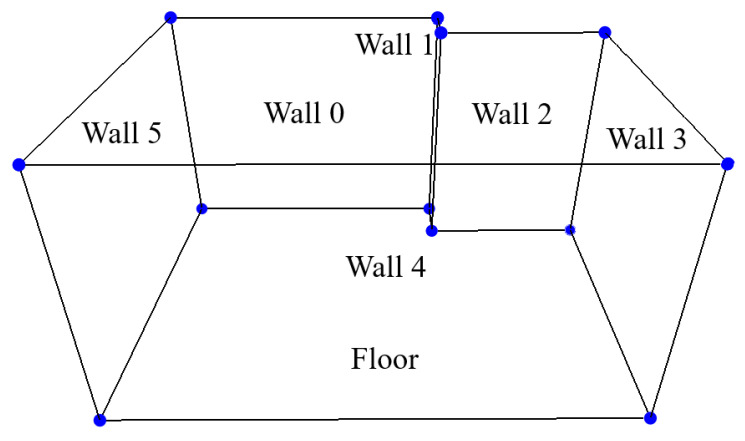
Result of the layout estimation step of VET.

From this step, VET extracts the 3D points that belong to the scene’s layout and can conduct the next step of the stage, layout estimation. Using the RSPD method and eliminating the duplicated planes, VET obtains the layout of the scene in about 30 s. The generated layout for the previously shown scene is depicted in Figure 12. This image shows the outline of the detected walls along with the corners where the walls intersect, creating a complete layout for the virtual scene.

**Figure 13:**
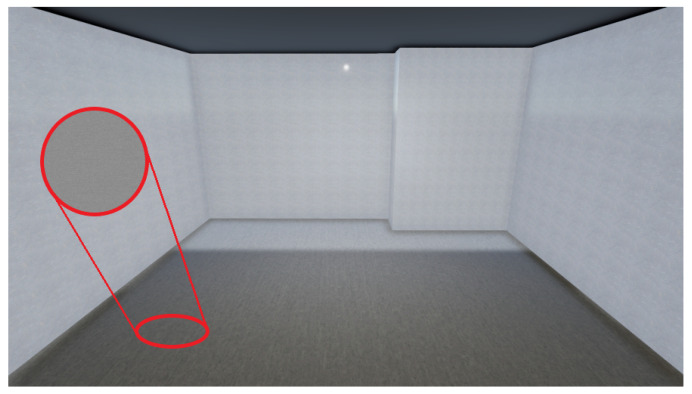
Result of the texture generation step of VET.

As a final step for the room limits extraction, VET generates textures for the planes (walls and floor). This method first extracts rectified crops of the planes from the images taken in the scanning of the room; then, the crops are fed into the plan2scene texture generation module, generating high-quality textures that resemble the real scene. Finally, the textures are loaded into the planes; the final result of a fully processed layout is presented in Figure 13, where we can observe that the generated textures are similar to the appearance of the original scene. This step takes approximately 2.5 min, including the crop selection and the generation of the textures.

**Figure 14:**
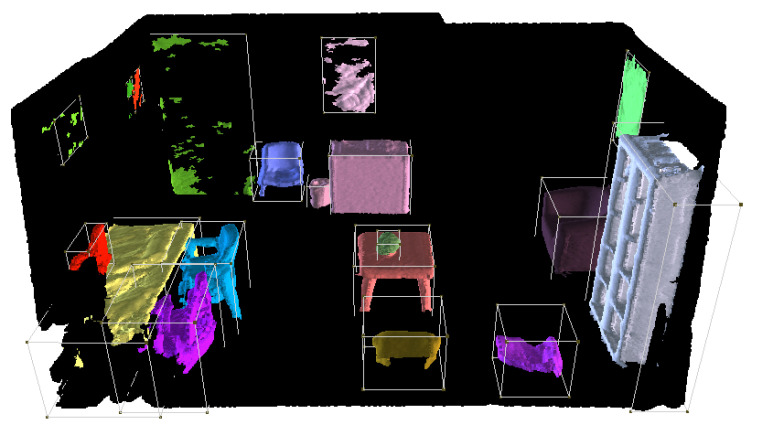
Result of the instance segmentation step of VET.

Simultaneously with the execution of the preceding steps, the process also advances on a parallel track focusing on the discrete objects within the scene. Similarly to the other stage, the first step is instance segmentation, where the aim is to obtain the individual instances inside the scene alongside a label that determines the class of the instance (each chair, table, cabinet, etc.). For this step, VET implements Mask3D, an SoA method for the task of instance segmentation, obtaining an Avg AP50 =0.780 on the ScanNet dataset and an Avg AP50 =0.388 on the ScanNet200 dataset. This metric measures the precision of a model’s predictions when considering the top 50% of the retrieved objects ranked by confidence scores. Regarding the completion time, it is dependent on the size of the scene and the point cloud; for the scene used in the previous figures, it takes approximately 3.5 min. The efficiency of this method can be seen in Figure 14, where the results of processing the 3D reconstruction with this method can be seen. Specifically, this step is able to correctly and individually detect the main objects of the scene, and assign them a unique color. The detected classes for this scene are chair, table, cabinet, bookshelf, trash can, door, armchair, coffee table, and monitor, some of which are exclusive of the ScanNet200 dataset.

With the instances detected and labeled, VET can carry out the CAD retrieval and alignment step, where the algorithm replaces the objects in the point cloud with the most similar CAD model in the given dataset and aligns it to position it in the scene. This step is carried out with an automatic version of ScanNotate, which outperforms the Scan2CAD approach, as stated in the original paper [17]. Additionally, we implemented an optional preselection of CAD models in an effort to reduce the computational costs of the original method. The computational cost of this method differs significantly depending on the number of instances in the scene and their class, classes such as chairs and tables with a high number of possible CAD models take the longest time, which is why we incorporated the faster method for these two classes. Specifically, for the scene used in the figures, the fast implementation with the refined dataset takes approximately 50 min, while the base implementation of ScanNotate with the whole ShapeNet dataset takes about 68 min.

**Figure 15:**
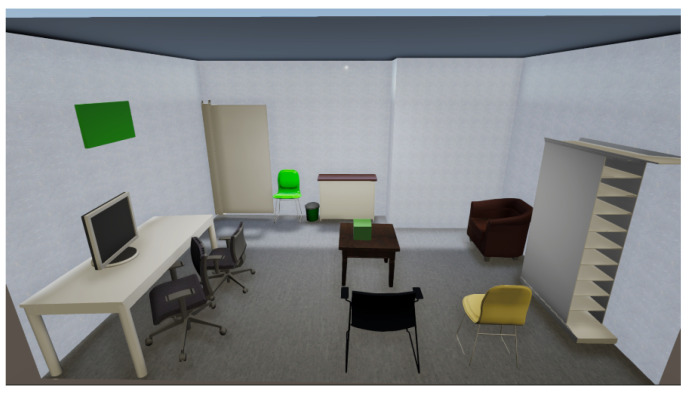
Reconstructed scene in the VR scenario.

Finally, by merging the textured layout extracted from the scene and the aligned CAD models, VET loads the scene into a VR application where the user has full interaction with the scene and a high level of editing power, being able to manipulate the objects, change the CAD model, and edit its appearance. Figure 15 shows the complete rendered scene, where we can see examples of the non-substituted objects represented with green cubes. As mentioned in the previous section, these objects can be substituted with CAD models or images (only for pictures) loaded by the user; alternatively, the user can select a different class CAD model of our database or eliminate them. Combining all the steps, the time to create the shown virtualization of the scene once the acquisition step has finished is approximately 57.5 min, thanks to the parallel execution of two stages. Specifically, this time was performed using a custom-built PC with an NVIDIA (Nvidia Corporation, Santa Clara, CA, USA, https://www.nvidia.com/ (accessed on 30 May 2024)) GeForce RTX 3060 12 GB, an AMD (Advanced Micro Devices, Inc., Santa Clara, CA, USA, https://www.amd.com/ (accessed on 30 May 2024)) Ryzen 7 5700 G, and 32 GB of DDR4 RAM. Breaking down the time step-by-step, once the scene is scanned by the user, the initial 3D reconstruction takes approximately 4 min, and the scene understanding step, which executes two sub-steps, takes the remaining 53.5 min. In this step, the CAD retrieval and alignment take the longest, and it also varies notably according to the contents of the scene. The implemented method is a brute force algorithm that simulates all of the possible CAD models for the given class. Therefore, the computational cost of the generation of the room is heavily influenced by the number and type of objects. Additionally, the time of the CAD retrieval and alignment step escalates linearly with the number of objects since the method processes them individually. The performance of this step increases drastically with the increase in the GPU computational power and the VRAM available. Regarding hardware and software requirements, it is necessary to use an NVIDIA GPU compatible with CUDA version 11.7, with at least 12 GB of VRAM to load the models. As for the CPU, it is recommended that it matches the computational power of the GPU to avoid bottlenecks. Finally, the framework is designed to be used in Windows 10.

**Figure 16:**
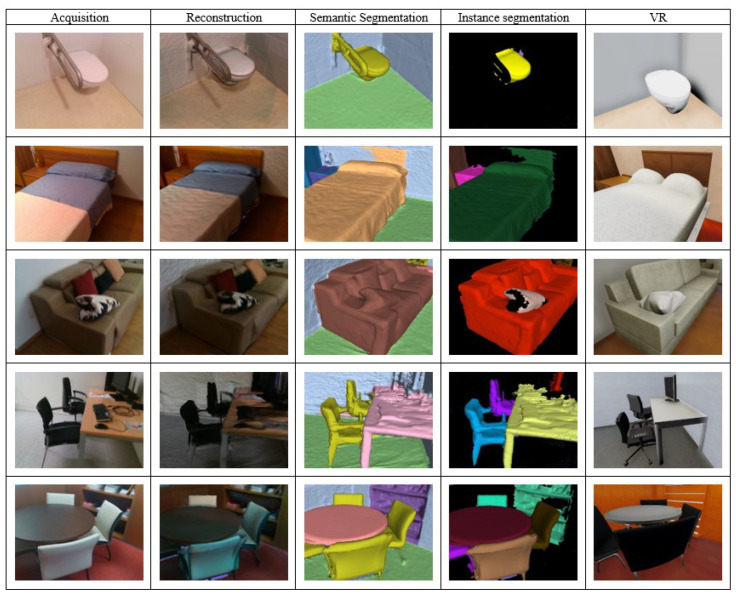
Results of multiple scenes virtualized with VET.

In the culmination of our discussion on VET, Figure 16 serves as a comprehensive showcase, illustrating the tool’s process across five distinct scenes that we have captured. Specifically, from top to bottom, the first scene is a bathroom, the second is a bedroom, the third is a living room, and the fourth and fifth scenes are two different offices. This figure vividly outlines the key phases of VET, encompassing layout extraction, texturing, CAD retrieval, and alignment, all culminating in the creation of the final virtual reality (VR) environment. It is important to note that the images were generated on a PC, but the VR column depicts what the user would see through a VR headset.

VET’s adaptability is highlighted through its application to a diverse array of room types, including bathrooms, bedrooms, living rooms, and offices. The figure not only demonstrates VET’s versatility but also evidences the high quality of the reconstructions, which faithfully mirror the original scenes. Through the lens of semantic segmentation, we discern VET’s proficiency in accurately delineating the architectural elements (notably, walls and floors) from furnishings and other objects (such as chairs and tables).

Further examination of the instance segmentation phase reveals VET’s adeptness at identifying and classifying each element within the scene across a broad spectrum of categories, effectively handling complex arrangements like groups of chairs around a table. The final VR visualization underscores VET’s ability to translate these meticulously reconstructed and segmented scenes into immersive, lifelike, and fully interactive virtual environments that closely replicate real-world settings.

This comprehensive depiction underscores VET’s integral role in advancing the field of scene reconstruction, offering a powerful tool for creating detailed and interactive models of real-world spaces.

### 4.1. Applications

This framework is developed as part of the EXPERIENCE project (EXPERIENCE PROJECT, Grant Agreement No. 101017727 Experience, https://experience-project.eu/ (accessed on 30 May 2024)), a European collaboration project involving multiple universities. The project receives funding from the European Union’s Horizon 2020 research and innovation program. EXPERIENCE’s goal is to obtain a new framework for the classification and treatment of the clinical spectrum of psychiatric disorders, unveiling previously undiscovered subtypes of anxiety, depression, and eating disorders through the use of VR scenarios.

VET plays an important role in the development of EXPERIENCE since it provides the capability of generating realistic and immersive 3D scenarios for non-expert users (i.e., psychologists). The 3D scenes generated with VET have multiple applications in the EXPERIENCE project; for instance, VET allows the reconstruction of familiar spaces in order to help the patients approach traumatic or phobic situations in a safe and controlled space, and in a gradual manner. The patients can experience new realities without feeling threatened through edited 3D scenes or non-familiar scenarios. Moreover, the patients’ safe spaces can be recreated and visited in different moods, with the addition of music and modifications in the lighting. Those scenes can be edited in many ways to study the patient’s capability of detecting the changes and the effects it has on them, among other implementations.

Furthermore, VET presents an opportunity in other fields, such as home decoration, where users could experience through VR how their home would look with specific furniture that stores provide; additionally, the client could reconfigure the furniture of the room to the desired distribution, saving time and effort later. Similarly, home renovations could be previewed in a VR experience, helping clients decide the renovations they want and creating a better experience for them. Similarly, real estate companies could offer a VR tour through the different properties they have to save time for both the company and the clients by giving them an immersive preview of the properties instead of visiting them all. Other applications can be found in home insurance, providing the insurance company with a digital representation of our homes, which would facilitate the estimation when calculating damages at our homes. Moreover, VET provides a cheap and easy way to generate 3D scenes for gaming and VR applications.

### 4.2. Limitations

In this subsection, we discuss the limitations of our automatic 3D scene generation framework. Despite its advancements and capabilities, it is essential to acknowledge the boundaries within which the framework operates. Understanding these limitations is crucial for interpreting the results and identifying areas for future improvement and development. The specific limitations of the methods implemented in VET have been pointed out in Section 3. However, this subsection provides a more in-depth analysis of the limitations, along with the general restraints of the framework.

**Figure 17:**
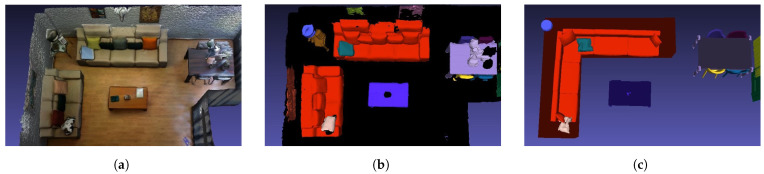
Example of wrong prediction of Mask3D. (**a**) 3D reconstruction. (**b**) Instance segmentation. (**c**) CAD replacement.

In the first step, the data acquisition, BundleFusion is sensitive to fast and sharp movements, and to reflective surfaces, since it might result in a temporary loss of the camera pose for some frames, rendering them unusable for posterior methods. However, the object can be partially reconstructed with frames that did not lose the tracking and substituted with a similar model, such as the monitor that can be seen in the sample scene in the results section (Section 4). Moreover, transparent objects present some difficulties, mainly the fact that the acquisition method does not detect transparent surfaces, and they will not show in the initial reconstruction. On a different note, highly detailed structures or objects can become less detailed if the elements are smaller than 1 cm (the voxel size for the reconstruction); however, most of the details can be represented with that voxel size, such as the individual books in a bookshelf or details in a poster, such as in Figure 10. Nonetheless, the model retrieved for highly detailed objects heavily depends on the dataset, and the objects could be replaced with more basic representations. The Mask3D method implemented in the instance segmentation step has some limitations in its predictions; mainly, if similar objects are close by in the 3D space, Mask3D may predict only one instance for multiple objects. For example, in Figure 17, the algorithm predicts only one instance for the two sofas, as it can be easily confused with an L-shaped sofa. Figure 17a shows the initial 3D reconstruction of the room, where we can see that there are two sofas; Figure 17b shows the instant segmentation results, in which we can see that the two sofas have been assigned the same color, thus belonging to the same instance; finally, Figure 17c shows the CAD replacement, which uses an L-shaped sofa to substitute the instance.

Moreover, the ScanNotate method implements a brute force algorithm that simulates every CAD model available for the class of the object being processed; thus, the computational cost is elevated. However, VET solves this limitation by implementing a faster mode that preselects a reduced list of possible CAD models for each object, achieving a more efficient implementation. Regarding the layout method, given how the planes are detected and processed to eliminate duplicates, the method is subjected to the Manhattan World assumption, meaning that the method is not suited for diagonal or circular walls; however, it can work with slightly non-parallel walls. Additionally, it may fail to detect partially scanned walls or walls with big gaps. Finally, the texture synthesis method generates the textures based on the input crop of the acquired images. Therefore, the lighting conditions can drastically change the generated texture, and the quality of the RGB image also influences the quality of the synthesized texture.

Nonetheless, most of these limitations can be compensated in the final visualization step of VET, where the user can explore the generated VR scenario and has the editing freedom to change the scene to match the necessities, enhancing the reliability of our framework.

On a more general note, the framework is limited by the initial input of the data acquisition step. Therefore, it is recommended that the user uses a good quality RGB-D camera, and it is important that the user scans the room carefully and in detail in order to ensure that the room has been correctly captured. Moreover, VET is tailored to specific indoor scenarios, specifically homes and offices, and will generate lower-quality virtualizations in industrial or sanitary environments. This is primarily due to the use of ScanNet for the training of Mask3D, which is focused on homes and offices, and the use of ShapeNet in the CAD alignment and retrieval step, which lacks CAD models for industrial or sanitary objects. Additionally, it is not targeted to dynamic scenarios, where objects, persons, or animals are in motion; this is due to the tracking method using the RGB and depth information to track the camera, and moving objects appear in different positions in each frame, possibly generating an incorrect camera pose prediction or simply losing completely the pose tracking. Finally, regarding scalability, with the hardware described in Section 4 and a voxel size of 1 cm, we were able to capture approximately 15,000 images in around 20 min, covering a surface area of roughly 75 m^2^; we recommend this value as a maximum surface area to ensure quality scanning of the room.

## 5. Conclusions

In conclusion, VET is a novel framework integrated into a single application that automates the complete virtualization of 3D scenes using CV and DL methods that have been quantitatively evaluated in specialized datasets. It is versatile, capable of handling diverse indoor scenes and capable of working with 200 object classes. This approach implements most of the current state-of-the-art methods for each pipeline step, generating an accurate virtualization of the 3D scene. This framework has been qualitatively analyzed using a variety of indoor scenes from a ScanNet-like custom dataset.

Future work includes training Mask3D to segment walls and floors, using these predictions to extract the layout information. Also, Mask3D could be retrained with industrial or sanitary scenes to improve the performance in those settings. Additionally, the CAD model dataset could be complemented with industrial or sanitary objects to further enhance the applicability of VET in those scenarios. Regarding the illumination of the scene, the light points could be generated in the same points as in the original scene, adding an extra step in the acquisition to annotate the pose of the lights. Furthermore, this framework could be extended to outdoor scenes, contributing to outdoor VR applications, which remains a challenge. Moreover, we plan to conduct an SUS usability test to substantiate the user-friendly claims of the framework. Finally, this framework will be applied in several real-use cases from the EXPERIENCE project, introduced in Section 4.1, further contributing to the usability claims of the framework since it will be used by non-expert users.

## Figures and Tables

**Table 1:** Comparison of state-of-the-art virtualization methods and VET.

Framework	3D Reconstruction		Layout Estimation		3D Object Understanding			Automated	CAD Align	Appearance	Scenario
	Method	Sensor	Input Data	Method	Input Data	Method	Amount of Classes				
**RealitySkins [14]**	KinectFusion	Kinect Structure Tango	RGB-D image	Hough transform	3D information	Random Forest Classifier	4	Yes	Occupancy grid loss	No	Indoor Scenarios
**Snap2CAD [10]**	Android Capture System	Android + RGB camera	-	-	RGB Image	HRNet-W48	13	Yes	CNN + PoseFromShape	No	Indoor Scenarios
**TransforMR [11]**	-	-	-	-	RGB Images	SMOKE	2	Yes	Custom method	No	Oudoor Scenarios
**GeoRec [15]**	GeoRec	Not Specified	RGB-D images	GeoLE	RGB-D images	GeoOD	9	Yes	Mesh Alignment	No	Indoor Scenarios
**VRFromX [4]**	Not Specified	LiDAR	-	-	-	Manual	-	No	Custom CNN + PointNetLK	No	Indoor Scenarios
**ScanToVR [3]**	-	-	Point Cloud	RANSAC	Point Cloud	SoftGroup	20	Yes	4D Spatio Temporal ConvNets	No	Indoor Scenarios
**OpenRooms [42]**	-	-	Point Cloud	RANSAC	Point Cloud	PartNet	24	Yes	Photoshape++	Yes	Indoor Scenarios
**VET**	BundleFusion	Intel RealSense D4XX	Point Cloud	Own	Point Cloud	Mask3D	200	Fully-automated	ScanNotate	Yes (planes)	Indoor Scenarios

**Table 2:** mIoU for layout classes evaluated on ScanNet dataset [18].

Classes	Wall	Floor	Cabinet	Door	Window
**mIoU**	0.868	0.958	0.770	0.640	0.744

## Data Availability

The necessary data to reconstruct the scenes in our dataset, along with the step-by-step results of the processing of the scene used during the results section, are available at https://github.com/Pamogar/VET-IndoorDataset (accessed on 30 May 2024).

## Abbreviations

The following abbreviations are used in this manuscript:

MDPI	Multidisciplinary Digital Publishing Institute
VR	Virtual Reality
CV	Computer Vision
DL	Deep Learning
VET	Virtual Experience Toolkit
SLAM	Simultaneous Localization and Mapping
CAD	Computer-Aided Design
GUI	Graphical User Interface
RSPD	Robust Statistics-based Plane Detection
O-CNN	Octree-based Convolutional Neural Networks
NeRF	Neural Radiance Fields
CNN	Convolutional Neural Networks
FCNN	Fully Connected Neural Networks
HRNet	High-Resolution Networks
RCNN	Region-based Convolutional Neural Network
DoF	Degrees of Freedom
ROI	Region Of Interest
ICP	Iterative Closest Point
SoA	State of the Art
UPV	Universitat Politècnica de Valencia
FCN	Fully Convolutional Networks

## References

[1] Zheng J., Chan K., Gibson I. (1998). Virtual reality. IEEE Potentials.

[2] Yang M.J., Guo Y.X., Zhou B., Tong X. Indoor scene generation from a collection of semantic-segmented depth images. Proceedings of the IEEE/CVF International Conference on Computer Vision.

[3] Kumar H.G., Khargonkar N.A., Prabhakaran B. (2018). ScanToVR: An RGB-D to VR Reconstruction Framework. https://bpb-us-e2.wpmucdn.com/sites.utdallas.edu/dist/f/1052/files/2023/03/final_draft_withnames.pdf.

[4] Ipsita A., Li H., Duan R., Cao Y., Chidambaram S., Liu M., Ramani K. VRFromX: From scanned reality to interactive virtual experience with human-in-the-loop. Proceedings of the Extended Abstracts of the 2021 CHI Conference on Human Factors in Computing Systems.

[5] Zhang Y., Devalapalli S., Mehta S., Caspi A. (2023). OASIS: Automated Assessment of Urban Pedestrian Paths at Scale. arXiv.

[6] Marullo G., Zhang C., Lamberti F. Automatic generation of affective 3D virtual environments from 2D images. Proceedings of the GRAPP.

[7] Simeone A.L., Velloso E., Gellersen H. Substitutional reality: Using the physical environment to design virtual reality experiences. Proceedings of the 33rd Annual ACM Conference on Human Factors in Computing Systems.

[8] Estrada J.G., Simeone A.L. Recommender system for physical object substitution in VR. Proceedings of the 2017 IEEE Virtual Reality (VR).

[9] Pintore G., Mura C., Ganovelli F., Fuentes-Perez L., Pajarola R., Gobbetti E. (2020). State-of-the-art in automatic 3D reconstruction of structured indoor environments. Computer Graphics Forum.

[10] Manni A., Oriti D., Sanna A., De Pace F., Manuri F. (2021). Snap2cad: 3D indoor environment reconstruction for AR/VR applications using a smartphone device. Comput. Graph..

[11] Kari M., Grosse-Puppendahl T., Coelho L.F., Fender A.R., Bethge D., Schütte R., Holz C. Transformr: Pose-aware object substitution for composing alternate mixed realities. Proceedings of the 2021 IEEE International Symposium on Mixed and Augmented Reality (ISMAR).

[12] Schult J., Engelmann F., Hermans A., Litany O., Tang S., Leibe B. (2022). Mask3D for 3D semantic instance segmentation. arXiv.

[13] Luo C., Zou B., Lyu X., Xie H. Indoor scene reconstruction: From panorama images to cad models. Proceedings of the 2019 IEEE International Symposium on Mixed and Augmented Reality Adjunct (ISMAR-Adjunct).

[14] Shapira L., Freedman D. Reality skins: Creating immersive and tactile virtual environments. Proceedings of the 2016 IEEE International Symposium on Mixed and Augmented Reality (ISMAR).

[15] Huan L., Zheng X., Gong J. (2022). GeoRec: Geometry-enhanced semantic 3D reconstruction of RGB-D indoor scenes. ISPRS J. Photogramm. Remote Sens..

[16] Cheng L.P., Ofek E., Holz C., Wilson A.D. Vroamer: Generating on-the-fly VR experiences while walking inside large, unknown real-world building environments. Proceedings of the 2019 IEEE Conference on Virtual Reality and 3D User Interfaces (VR).

[17] Ainetter S., Stekovic S., Fraundorfer F., Lepetit V. Automatically annotating indoor images with CAD models via RGB-D scans. Proceedings of the IEEE/CVF Winter Conference on Applications of Computer Vision.

[18] Dai A., Chang A.X., Savva M., Halber M., Funkhouser T., Nießner M. Scannet: Richly-annotated 3D reconstructions of indoor scenes. Proceedings of the IEEE Conference on Computer Vision and Pattern Recognition.

[19] Dai A., Nießner M., Zollhöfer M., Izadi S., Theobalt C. (2017). Bundlefusion: Real-time globally consistent 3D reconstruction using on-the-fly surface reintegration. ACM Trans. Graph..

[20] Rozenberszki D., Litany O., Dai A. (2022). Language-grounded indoor 3D semantic segmentation in the wild. Proceedings of the European Conference on Computer Vision.

[21] Araújo A.M., Oliveira M.M. (2020). A robust statistics approach for plane detection in unorganized point clouds. Pattern Recognit..

[22] Wang P.S., Liu Y., Guo Y.X., Sun C.Y., Tong X. (2017). O-cnn: Octree-based convolutional neural networks for 3D shape analysis. ACM Trans. Graph..

[23] Garcia C., Mora P., Ortega M., Ivorra E., Valenza G., Alcañiz M.L. Virtual experience toolkit: Enhancing 3D scene virtualization from real environments through computer vision and deep learning techniques. Proceedings of the 2023 IEEE International Conference on Metrology for eXtended Reality, Artificial Intelligence and Neural Engineering (MetroXRAINE).

[24] Linder W. (2009). Digital Photogrammetry.

[25] Han M., Zhang Z., Jiao Z., Xie X., Zhu Y., Zhu S.C., Liu H. (2022). Scene reconstruction with functional objects for robot autonomy. Int. J. Comput. Vis..

[26] Yang H., Zhang H. Efficient 3D room shape recovery from a single panorama. Proceedings of the IEEE Conference on Computer Vision and Pattern Recognition.

[27] Yang Y., Jin S., Liu R., Kang S.B., Yu J. Automatic 3D indoor scene modeling from single panorama. Proceedings of the IEEE Conference on Computer Vision and Pattern Recognition.

[28] Izadi S., Kim D., Hilliges O., Molyneaux D., Newcombe R., Kohli P., Shotton J., Hodges S., Freeman D., Davison A. Kinectfusion: Real-time 3D reconstruction and interaction using a moving depth camera. Proceedings of the 24th Annual ACM Symposium on User Interface Software and Technology.

[29] Prisacariu V.A., Kähler O., Golodetz S., Sapienza M., Cavallari T., Torr P.H., Murray D.W. (2017). Infinitam v3: A framework for large-scale 3D reconstruction with loop closure. arXiv.

[30] Whelan T., Leutenegger S., Salas-Moreno R.F., Glocker B., Davison A.J. ElasticFusion: Dense SLAM without a pose graph. Proceedings of the Robotics: Science and Systems.

[31] Wang J., Wang P., Long X., Theobalt C., Komura T., Liu L., Wang W. (2022). Neuris: Neural reconstruction of indoor scenes using normal priors. Proceedings of the European Conference on Computer Vision.

[32] Gao Y., Cao Y.P., Shan Y. SurfelNeRF: Neural surfel radiance fields for online photorealistic reconstruction of indoor scenes. Proceedings of the IEEE/CVF Conference on Computer Vision and Pattern Recognition.

[33] Hedau V., Hoiem D., Forsyth D. Recovering the spatial layout of cluttered rooms. Proceedings of the 2009 IEEE 12th International Conference on Computer Vision.

[34] Dasgupta S., Fang K., Chen K., Savarese S. Delay: Robust spatial layout estimation for cluttered indoor scenes. Proceedings of the IEEE Conference on Computer Vision and Pattern Recognition.

[35] Lee C.Y., Badrinarayanan V., Malisiewicz T., Rabinovich A. Roomnet: End-to-end room layout estimation. Proceedings of the IEEE International Conference on Computer Vision.

[36] Zou C., Colburn A., Shan Q., Hoiem D. Layoutnet: Reconstructing the 3D room layout from a single rgb image. Proceedings of the IEEE Conference on Computer Vision and Pattern Recognition.

[37] He Y., Yu H., Liu X., Yang Z., Sun W., Wang Y., Fu Q., Zou Y., Mian A. (2021). Deep learning based 3D segmentation: A survey. arXiv.

[38] Moro S., Komuro T. (2021). Generation of virtual reality environment based on 3D scanned indoor physical space. Proceedings of the Advances in Visual Computing: 16th International Symposium, ISVC 2021.

[39] Xiao Y., Qiu X., Langlois P.A., Aubry M., Marlet R. (2019). Pose from shape: Deep pose estimation for arbitrary 3D objects. arXiv.

[40] Aoki Y., Goforth H., Srivatsan R.A., Lucey S. Pointnetlk: Robust & efficient point cloud registration using pointnet. Proceedings of the IEEE/CVF Conference on Computer Vision and Pattern Recognition.

[41] Akl A., Yaacoub C., Donias M., Da Costa J.P., Germain C. (2018). A survey of exemplar-based texture synthesis methods. Comput. Vis. Image Underst..

[42] Li Z., Yu T.W., Sang S., Wang S., Song M., Liu Y., Yeh Y.Y., Zhu R., Gundavarapu N., Shi J. Openrooms: An open framework for photorealistic indoor scene datasets. Proceedings of the IEEE/CVF Conference on Computer Vision and Pattern Recognition.

[43] Yeh Y.Y., Li Z., Hold-Geoffroy Y., Zhu R., Xu Z., Hašan M., Sunkavalli K., Chandraker M. Photoscene: Photorealistic material and lighting transfer for indoor scenes. Proceedings of the IEEE/CVF Conference on Computer Vision and Pattern Recognition.

[44] Yan K., Luan F., Hašan M., Groueix T., Deschaintre V., Zhao S. Psdr-room: Single photo to scene using differentiable rendering. Proceedings of the SIGGRAPH Asia 2023 Conference Papers.

[45] Li X., Dong Y., Peers P., Tong X. (2017). Modeling surface appearance from a single photograph using self-augmented convolutional neural networks. ACM Trans. Graph..

[46] Dong W., Lao Y., Kaess M., Koltun V. (2022). ASH: A modern framework for parallel spatial hashing in 3D perception. IEEE Trans. Pattern Anal. Mach. Intell..

[47] Garland M., Heckbert P.S. Simplifying surfaces with color and texture using quadric error metrics. Proceedings of the IEEE Visualization ’98 (Cat. No. 98CB36276).

[48] Kadambi A., Bhandari A., Raskar R. (2014). 3D Depth Cameras in Vision: Benefits and Limitations of the Hardware: With an Emphasis on the First-and Second-Generation Kinect Models. Computer Vision and Machine Learning with RGB-D Sensor.

[49] Li Y., Li W., Tang S., Darwish W., Hu Y., Chen W. (2020). Automatic indoor as-built building information models generation by using low-cost RGB-D sensors. Sensors.

[50] Armeni I., Sener O., Zamir A.R., Jiang H., Brilakis I., Fischer M., Savarese S. 3D semantic parsing of large-scale indoor spaces. Proceedings of the IEEE Conference on Computer Vision and Pattern Recognition.

[51] Chen M., Hu Q., Yu Z., Thomas H., Feng A., Hou Y., McCullough K., Ren F., Soibelman L. (2022). STPLS3D: A Large-Scale Synthetic and Real Aerial Photogrammetry 3D Point Cloud Dataset. arXiv.

[52] Chang A.X., Funkhouser T., Guibas L., Hanrahan P., Huang Q., Li Z., Savarese S., Savva M., Song S., Su H. (2015). Shapenet: An information-rich 3D model repository. arXiv.

[53] Wu Z., Song S., Khosla A., Yu F., Zhang L., Tang X., Xiao J. 3D shapenets: A deep representation for volumetric shapes. Proceedings of the IEEE Conference on Computer Vision and Pattern Recognition.

[54] Beyer T., Dai A. (2022). Weakly-supervised end-to-end cad retrieval to scan objects. arXiv.

[55] Vidanapathirana M., Wu Q., Furukawa Y., Chang A.X., Savva M. Plan2scene: Converting floorplans to 3D scenes. Proceedings of the IEEE/CVF Conference on Computer Vision and Pattern Recognition.

[56] Ren T., Liu S., Zeng A., Lin J., Li K., Cao H., Chen J., Huang X., Chen Y., Yan F. (2024). Grounded SAM: Assembling Open-World Models for Diverse Visual Tasks. arXiv.

[57] Henzler P., Mitra N.J., Ritschel T. Learning a neural 3D texture space from 2d exemplars. Proceedings of the IEEE/CVF Conference on Computer Vision and Pattern Recognition.

